# Aberrant MCM10 SUMOylation induces genomic instability mediated by a genetic variant associated with survival of esophageal squamous cell carcinoma

**DOI:** 10.1002/ctm2.485

**Published:** 2021-06-27

**Authors:** Jianbo Tian, Zequn Lu, Siyuan Niu, Shanshan Zhang, Pingting Ying, Lu Wang, Ming Zhang, Yimin Cai, Tianyi Dong, Ying Zhu, Rong Zhong, Zhihua Wang, Jiang Chang, Xiaoping Miao

**Affiliations:** ^1^ Department of Epidemiology and Biostatistics Key Laboratory for Environment and Health School of Public Health Tongji Medical College Huazhong University of Sciences and Technology Wuhan China; ^2^ Department of Urology Tongji Hospital Tongji Medical College Huazhong University of Science and Technology Wuhan China

**Keywords:** ESCC, genomic instability, MCM10, SUMOylation, survival

## Abstract

**Background:**

Esophageal squamous cell carcinoma (ESCC) is one of the common gastrointestinal malignancy with an inferior prognosis outcome. DNA replication licensing aberration induced by dysregulation of minichromosome maintenance proteins (MCMs) causes genomic instability and cancer metastasis. SUMOylation modification plays a pivotal role in regulation of genomic integrity, while its dysregulation fueled by preexisting germline variants in cancers remains poorly understood.

**Methods:**

Firstly, we conducted two‐stage survival analysis consisting of an exome‐wide association study in 904 ESCC samples and another independent 503 ESCC samples. Then, multipronged functional experiments were performed to illuminate the potential biological mechanisms underlying the promising variants, and MCM10 influences the ESCC progression. Finally, we tested the effects of MCM10 inhibitors on ESCC cells.

**Results:**

A germline variant rs2274110 located at the exon 15 of MCM10 was identified to be significantly associated with the prognosis of ESCC patients. Individuals carrying rs2274110‐AA genotypes confer a poor survival (hazard ratio = 1.61, 95% confidence interval = 1.35–1.93, *p *= 1.35 × 10^−7^), compared with subjects carrying rs2274110‐AG/GG genotypes. Furthermore, we interestingly found that the variant can increase SUMOylation levels at K669 site (Lys[K]699Arg[R]) of MCM10 protein mediated by SUMO2/3 enzymes, which resulted in an aberrant overexpression of MCM10. Mechanistically, aberrant overexpression of MCM10 facilitated the proliferation and metastasis abilities of ESCC cells in *vitro* and in *vivo* by inducing DNA over‐replication and genomic instability, providing functional evidence to support our population finding that high expression of MCM10 is extensively presented in tumor tissues of ESCC and correlated with inferior survival outcomes of multiple cancer types, including ESCC. Finally, MCM10 inhibitors Suramin and its analogues were revealed to effectively block the metastasis of ESCC cells.

**Conclusions:**

These findings not only demonstrate a potential biological mechanism between aberrant SUMOylation, genomic instability and cancer metastasis, but also provide a promising biomarker aiding in stratifying ESCC individuals with different prognosis, as well as a potential therapeutic target MCM10.

## Graphical abstract

1. A two‐stage survival study consisting of 1407 subjects finds a significant association between MCM10‐rs2274110 and ESCC survival.
2. The MCM10 variant can increase MCM10 SUMOylation levels and result in MCM10 aberrant overexpression, which facilitates ESCC cells proliferation and migration by inducing genomic instability.
3. The MCM10 inhibitors Suramin and its analogues can block ESCC cells metastasis.

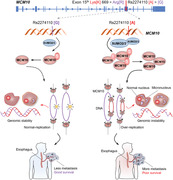

**Abbreviations**
CDC6: chromatin licensing and DNA replication factor 1
CDT1: origin recognition complex
CIs: confidence intervals
CMG: Cdc45‐Mcm2‐7‐GINS
Co‐IP: co‐immunoprecipitation
ESCC: esophageal squamous cell carcinoma
EWS: Ewing's sarcoma.
GEO: gene expression omnibus
GWAS: genome‐wide association study
HRs: hazard ratios
ICGC: International Cancer Genome Consortium cohort
LC‐MS/MS: liquid chromatography tandem mass spectrometry
MCM10: minichromosome maintenance 10 replication initiation factor
MCMs: minichromosome maintenance proteins
ORC: cell division cycle 6
PALB2: partner and localizer of BRCA2
PTMs: post‐translational modifications
qRT‐PCR: quantitative reverse transcription PCR
TCGA: The Cancer Genome Atlas

## 1 INTRODUCTION

Esophageal squamous cell carcinoma (ESCC) is one of the common gastrointestinal malignancy in China, and its clinical prognosis is poor with 5‐year survival < 30%.^1^ Surgery supplemented with chemotherapy or chemoradiotherapy was the standard treatment of ESCC. However, these treatments were accompanied by a considerable increase in health‐related quality of life and yet still a poor prognosis.^2^ Accordingly, effective prognostic markers or therapy targets need to be identified for this malignant cancer. Inherited genetic polymorphisms have been proved to influence their phenotypic differences, such as genetic susceptibility of disease, treatment responses, drug resistance, and clinical prognosis.^3^ Previously a genome‐wide association study (GWAS) has identified a regulatory variant in *SLC39A6* that may serve as a prognostic marker of ESCC.^4^ These findings indicate that preexisting germline variants are worthy to be investigated for identifying both effective prognostic markers and therapy targets for this cancer.

Genomic instability, a hallmark of human cancer, is the major driver of tumor evolution and also shown to be associated with poor clinical prognosis, metastasis, and therapeutic resistance of tumors.^5^, ^6^ The proper licensing of DNA replication origins is an essential event for timely duplication and stability of genome. This replication licensing system requires the stable loading of some replication licensing factors, such as origin recognition complex (ORC), chromatin licensing and DNA replication factor 1 (CDT1), and the recruitment of the minichromosome maintenance (MCM) complex at replication origins.^7^, ^8^ Errors or aberration in replication licensing system might result in genomic instability across evolution and cell development.^7^ It was reported that in normal cells, the licensing system of DNA replication usually protect cells from replicative stress and genomic instability.^9^, ^10^ However, in tumor cells, these factors were shown to be widely upregulated and were associated with cancer progression or poor clinical outcomes.^11^, ^12^, ^13^, ^14^ Depletion of the MCMs inhibited the tumor growth and progression, suggesting they may not only be prognostic markers, but play important roles in cancer development.^14^, ^15^ It was also reported that in pre‐malignant cells, the overexpression of replication licensing factors, such as CDC6 and CDT1, could facilitate malignant behavior conversion.^16^ Besides, the combined deregulation of CDC6 and CDT1 was also shown to lead to DNA re‐replication in progenitor cells and lethal tissue dysplasia.^17^, ^18^, ^19^ Therefore, these accumulating evidences provide a conjecture supporting that overexpression of these replication licensing factors, including MCMs families, in human cancer cells may cause DNA “over‐replication” stress and genomic instability, which might further deteriorate cancer progression and poor prognosis.

SUMOylation is one of post‐translational protein modification (PTM) and recognized by small ubiquitin‐like modifier (SUMO) and plays important roles in responding cellular stress, stabling genomic integrity, regulating transcriptional patterns of genes, and signal transduction pathways and thus which is essential for cell homeostasis in eukaryotes.^20^ Importantly, SUMO pathway components appear to be upregulated in many cancers.^21^ Tumor cells may indeed require this “SUMOylation activation” to maintain the compromised robustness or otherwise easily occur dysregulation of gene transcriptional expression patterns and pathological activation of cancer signaling pathways.^22^ Thus, in a potentially hostile microenvironment, SUMOylation would contribute substantially to facilitating proliferation and migration of cancer cells. Given that SUMOylation‐regulated mechanisms are essential for cells in mammals, loss‐of‐function caused by germline variants in recognition sites of the SUMOylation process might be a potential biological mechanism contributing to diseases development and prognosis.

Here, we firstly conducted a two‐stage survival analysis totally consisting of 1407 ESCC samples by using our previous exome‐chip data and Taqman genotyping.^23^ We identified a SUMOylation variant rs2274110 located at the 15th exon of *MCM10*, which confers an inferior prognosis of ESCC. Mechanistically, we found that this variant upregulates the expression of MCM10 protein by increasing its SUMOylation level mediated by SUMO2/3. Furthermore, we indicated that upregulation of MCM10 facilitates the metastasis of ESCC cells both by inducing genomic instability, which may be induced by the DNA over‐replication. Moreover, we also showed that the MCM10 inhibitors Suramin and its analogues (NF157, NF546, and PPADS) can be used as potential anti‐cancer agents for ESCC, but are warranted to deconstruct the precise mechanisms and effective targets of these drugs before its clinical use. Our data provide insights into the link between aberrant SUMOylation, genomic instability, and cancer.

## 2 METHODS

### 2.1 Subjects and variants genotyping

In the present study, a two‐stage survival analysis totally consisting of 1407 ESCC samples was performed, and the baseline characteristics of all samples were detailed in Table S1. The variants genotypes of samples were detecting by using the Illumina HumanExome Beadchip system in the discovery stage, which has been detailed described previously.^23^ Briefly, we enrolled a total of 904 ESCC patients in the discovery stage who had available genotype data of variants, detailed survival time and outcomes, smoking and drinking status and tumor stage, from the Cancer Hospital, Chinese Academy of Medical Sciences in Beijing, China. In addition, a total of 503 ESCC patients who were enrolled from multiple hospitals in Wuhan were genotyped with Taqman genotyping assay (ABI 7500HT System, Applied Biosystems, USA) in the replication stage. We applied medical records and interviews to collect baseline characteristics and clinical information of ESCC individuals. The survival time of ESCC in this study were determined as the number of days from the date of first diagnosis to the date of death or date of last known contact. Noticeably, written informed consent was collected from all participants, and this study was also approved by the institutional review board of Huazhong University of Science and Technology (HUST, [2016]IEC(S160)).

### 2.2 Cell lines

ESCC cell lines KYSE30 and KYSE150 obtained from the China Center for Type Culture Collection (Shanghai, China), were cultured with Dulbecco's modified eagle's medium, 10% fetal bovine serum and 1% antibiotics consisting of penicillin and streptomycin (Gibco, USA), respectively. Noticeably, KYSE30 and KYSE150 cell lines used here were by authenticated by short tandem repeat profiling (Applied Biosystems, USA) and validated for the absence of mycoplasma contamination (MycoAlert, USA).

### 2.3 Construction and transfections

For transient transfection assay, the full‐length cDNA of MCM10 consisting of rs2274110[A] allele (named as MCM10[A]) or rs2274110[G] allele (named as MCM10[G]) was commercially synthesized and inserted into the pcDNA3.1(+) vector (Invitrogen, USA) by Genewiz (Suzhou, China). For lentivirus production and transfection, the full‐length cDNA of MCM10[A] or MCM10[G] was inserted into the pLVX‐PGK‐Puro and rLV‐Luciferase‐Hygro vectors. The X‐tremeGENE9 reagent (Roche, USA) and Lenti‐XTM concentrator were used to produce Lentivirus in 293T cells. Lentivirus‐containing plasmids were transfected into ESCC KYSE30 and KYSE150 cells, and 2 mg/ml puromycin was used for antibiotic selection.

### 2.4 RNA interference and CRISPR/Cas9 system

The siRNAs targeting SUMO2/3 and shRNAs targeting MCM10 used for knockdown assays were synthesized by RiboBio company (Guangzhou, China). All sequences of oligonucleotides were listed in Table S2. The quantitative reverse transcription PCR (qRT‐PCR) and western blotting were conducted to evaluate the transfection effect (Figures S2A and S2B). Furthermore, we applied CRISPR‐Cas9 editing system (Genloci Biotechnologies, China) to knockout MCM10 in KYSE30 and KYSE150 cell lines with pGK1.1‐CRISPR‐Cas9 vector (Cat# GP0134). We designed the single guide RNAs (sgRNAs) targeting MCM10 using by the CRISPR design tool (http://crispr.mit.edu). The sgRNA sequence targeting MCM10 sites are detailed in Table S2, and the effects of MCM10 knockout in ESCC cells were determined by Western‐blotting assay (Figure S2C).

### 2.5 qRT‐PCR

TRIzol reagent (Applied Biosystems, USA) was applied to harvest total RNA of ESCC tissues or cell lines. Subsequently, SuperScriptIII First‐Strand Synthesis System (Invitrogen, USA) and Power SYBRTM Green PCR Master Mix (Applied Biosystems, USA) was used to conduct reverse transcription and quantitative PCR assays, respectively. Noticeably, the expression of *GAPDH* was used to normalize the expression of target genes. All specific qPCR primers used can be seen in Table S2.

### 2.6 Western blotting

Total protein of ESCC tissues or cell lines were extracted by RIPA lysis buffer supplemented with protease inhibitors PMSF (Beyotime, China), and PhosSTOP (Sigma, USA) and then followed by sonication homogenization. Protein was incubated with primary antibodies against SUMO2/3 (1:1,000; Cat# 4971, CST), MCM10 (1:1,000; ab3733, Abcam), and β‐actin (1:1,000; Cat# 60008‐1‐Ig, Proteintech, China) at 4°C overnight.

### 2.7 Co‐Immunoprecipitation

KYSE30 and KYSE150 cells were cultured in 15 cm cell plate and firstly transfected with pcDNA‐Ctrl, MCM10[A] or MCM10[G] plasmids. After 48 h transfection, ESCC cells were lysed with a RIPA buffer supplement with protease inhibitors. For co‐immunoprecipitation (CO‐IP) experiments, 10 μg MCM10 antibody (ab3733, Abcam) or SUMO2/3 antibody (Cat # 4971, CST) was incubated with 1 mg cellular protein at 4°C for 3 h. Immunoprecipitates were captured and incubated with Protein A/G beads (20 μL; Cat# LSKMAGAG02, Millipore, USA) at 4°C overnight. Furthermore, the beads were washed extensively by lysis buffer for three times. Subsequently the elution was heated with 4 × loading buffer and were used to analyze by western blotting.

### 2.8 Liquid chromatography tandem mass spectrometry analysis

For beads samples, the preparation process of protein was adapted based on previously reported research.^24^ The wash buffer consisting of 100 mM Tris‐Cl and 150 mM NaCl was added and used to wash the IP beads binding with proteins. Furthermore, we added 50 μl of elution buffer consisting of 1% SDC/100 mM Tris‐Cl, 10 mM TCEP, and 40 mM CAA to elute protein and followed by reduction and alkylation reaction at 95°C for 10 min. Subsequently, we used the self‐made SDB tip columns to desalt the peptide. Finally, the protein samples were dried by vacuum and stored at ‐80°C until MS measurement. Liquid chromatography tandem mass spectrometry (LC‐MS/MS) detection was conducted with hybrid quadrupole‐TOF mass spectrometer (TripleTOF 5600+, SCIEX, USA) equipped with a nanoLC system (Eksigent).^25^ Detailed LC/MS processes had been described in our previous research.^26^ The ProteinPilot Software (SCIEX, MA, USA) was applied to search and identify proteins and UniProt human proteome as reference.^27^ Variable modifications were assigned as SUMO2135 and SUMO3549 of lysine. Only high‐quality peptide assignments with more than 95% confidence can be selected for further analysis, and the *p* < 0.05 was determined as the statistically significant.

### 2.9 Cell proliferation and migration

For the cell proliferation assay, cells were digested by trypsin, and 100 μl of cell suspension counted 2.5 × 10^2^ cells were seeded in 96‐well plate. After incubation for 24, 48, 72, and 96 h, we detected the absorbance at 450 nm to reflect the cell viability using CCK‐8 assay kit (Dojindo, Japan). For the cell migration assay, the cells were firstly starved with serum‐free medium for 8 h. Then, prior to the cell digestion, mitomycin C (10 μg/ml; Roche, Germany) was added into cells and incubated for 2 h. Cell suspension containing 2.5 × 10^3^ serum‐starved cells were placed and cultured in the upper chamber of Transwell Clear Polyester Membrane Inserts (Cat# 3422, 8‐μm pore filter, Corning Costar), while in the bottom chamber were added by culture media containing 20% FBS. After 24 h in culture, the migrated cells were fixed by 3.7% formaldehyde, permeabilized with 100% methanol and stained with 0.5% crystal violet, respectively. Then, the cells on upper surface of the Transwell chamber were scraped by a cotton swab, and the cells bottom surface of the Transwell filter were counted as the invasive cells. The number of migrated cells were examined by microscopy and counted in eight random areas.

### 2.10 Animal experiments

Female, aged 4–5 weeks BALB/c nude mice were purchased from the Vital River Laboratory Animal Technology (Beijing, China). For the lung metastasis model, we injected luciferase labeled ESCC cells (0.1 ml, 2 × 10^6^ cells) into nude mice via tail vein. Eight weeks after injection, the nude mice were injected with d‐luciferin intraperitoneally (100 μl, 30 mg/ml, Promega, USA) and reaction 10 min. Then, the nude mice were maintained under general anesthesia with isoflurane and further imaged by the IVIS system. The quantitation of lung metastases in vivo was measured by using an IVIS Lumina II (Caliper) equipped with Living Image software (PerkinElmer). Finally, we used paraformaldehyde to fix the lung tissues of nude mice and stain it with H&E. All experimental procedures were approved by the Institutional Animal Care and Use Committee of HUST [IACUC_Number:S2344].

### 2.11 Micronucleus assay

The micronucleus (MN) assay in ESCC cells was conducted using the In Vitro MicroFlow Kit (Litron, USA), which is a two‐color labeling technique. Briefly, KYSE30 and KSYE150 cells at a density of 2.5 × 10^4^ per well were seeded into six‐well cell plates. After 24 h transfection, Nucleic Acid Dye A (EMA) and Nucleic Acid Dye B (SYTOX Green) were added to stain compromised outer membrane and chromatin of ESCC cells, respectively. Subsequently, we applied Complete Lysis solution to digest these stained cells. Finally, we used flow cytometric (LSRII, BD Biosciences, USA) to analyze the proportion of two‐color labeling cells and calculated the differential staining between healthy chromatin and dead/dying cells.

Additionally, we also applied another method cytokinesis block^28^ to evaluate the MN proportion in ESCC cells or healthy individuals. For ESCC cells, cell suspension was harvested after 24 h transfection. For healthy individuals, each subject provided 0.5 ml of blood, which was supplemented with heparin and 4.5 ml of culture medium containing 15% fetal calf serum, penicillin (100 IU/ml) and phytohemagglutinin (20 μg/ml; Sigma, USA). Furthermore, the cell cultures were added into a cytochalasin B (6 μg/ml; Sigma, USA) and incubated for 3 days. Then, we prepared cell slides and stained cells with 10% Giemsa solution. Finally, we counted the proportion of micronucleated binucleated cells in 1000 binucleated lymphocytes, as well as micronucleated ESCC cells in 1000 tumor cells under a light‐microscopy.

### 2.12 The assays for testing the effect of MCM10 inhibitors on KYSE30 and KYSE150 cells

To assess the efficacy of several MCM10 inhibitors Suramin and its analogues (NF157, NF546 and PPADS)^29^, ^30^ on ESCC cells proliferation and migration abilities, we firstly determine the half‐maximal inhibitory concentration (IC_50_) of these inhibitors by measuring the cell viability at different time points. Drug treatment was started 24 h post‐seeding and continued for 48 h, and then ESCC cells were harvested and used for testing the cell viability. Furthermore, Suramin (Cat# HY‐B0879, MedChemExpress, USA), NF157, NF546, and PPADS (Cat# 2450, Cat# 3892 and Cat# 0625, R&D Systems, USA) at a concentration of IC_50,_ respectively, were added into ESCC KYSE30 and KYSE150 cells for incubation 48 h, and then cells were harvested and used for cell proliferation and migration testing.

### 2.13 Statistical analysis

The Cox regression analysis under an additive genetic model was conducted to calculate the associations of candidate germline variants with survival status of ESCC subjects with adjustment for some confounders, such as age, sex, smoking status, drinking status, and tumor stage. The comparation of overall survival of ESCC between groups with low MCM10 expression and high MCM10 expression was performed by Kaplan–Meier survival analysis and the corresponding *p* values, hazard ratios (HRs), and 95% confidence intervals (CIs) were determined by the log‐rank test. Besides, we used the paired Student's *t* test to compare the differences between both matched groups, and applied the Student's *t*‐test to test the differences between both non‐matched groups, respectively. The one‐way variance analysis was applied to compare the homogeneity of variance among the groups. For functional experiments, each experiment was performed independently three times, each with triplicates at least. All statistical calculation was conducted by R (3.3.0) or SPSS software (21.0).

## 3 RESULTS

### 3.1 MCM10 rs2274110‐AA genotypes confer an inferior survival of ESCC patients

**FIGURE 1:**
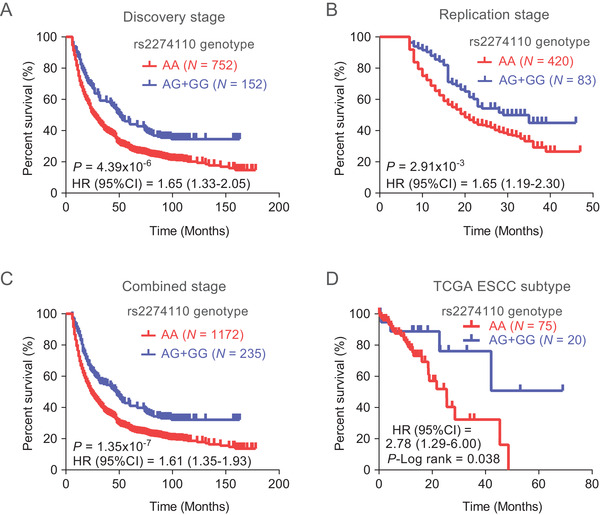
MCM10 rs2274110[A] is associated with the poor prognosis of ESCC. (A‐C) Kaplan‐Meier estimates of survival time for patients with ESCC stratified by MCM10‐rs2274110 genotype in discovery stage (A), replication stage (B), and combined stage (C) consisting of the discovery and replication stage. (D) Kaplan‐Meier estimates of survival time for patients with ESCC from TCGA dataset stratified by MCM10‐rs2274110 genotype. *p* values, HRs, and 95% CIs were calculated using Cox regression analysis with adjustments for age, sex, smoking status, drinking status, and tumor stage in (A‐C), and using log‐rank test in (D)

To identify promising prognostic biomarkers, we firstly carried out an exome‐wide association analysis with survival of 904 ESCC patients to identify candidate SNPs‐correlated with ESCC prognosis (Figure S1). The quantile‐quantile plot showed that there is a good match between the distributions of the observed *p* values and the expected ones by chance; and a small genomic control factor revealed a minimal inflation of genome‐wide association significance (λ = 1.052; Figure S1A). Strikingly, a missense variant rs2274110 located at the 15th exon of MCM10 was identified to confer a poor survival of ESCC patients with a promising association (Figure S1B). ESCC subjects carrying rs2274110‐AA genotypes tended to confer an inferior overall survival, compared to those with rs2274110‐AG/GG genotypes (*p *= 4.39 × 10^−6^, HR = 1.65, 95% CI = 1.33–2.05, Figure 1A). The association is successfully replicated in an independent cohort with 503 ESCC patients with *p* values being 2.91 × 10^−3^ (HR = 1.65, 95% CI = 1.19–2.30, Figure 1B). In addition, we further combined the results from both stages and still found that compared with individuals carrying the rs2274110‐AG/GG genotype, the patients with AA genotype present a shorter survival time of ESCC with HR being 1.61 (95% CI = 1.35–1.93, *p *= 1.35 × 10^−7^, Figure 1C). Consistently, these results are further validated in ESCC patients obtained from The Cancer Genome Atlas (TCGA) database (HR = 2.78, 95% CI = 1.29–6.00, *p *= 0.038, Figure 1D). Moreover, we also evaluated the association of rs2274110 genotypes with tumor stage of ESCC patients and interestingly found that rs2274110‐AA genotype carriers have a more advanced tumor stage, compared to the subjects with rs2274110‐AG/GG genotypes (Table S3). Taken together, these results provide population evidence supporting that ESCC patients carrying rs2274110‐AA genotype had an inferior prognosis and might harbor an aggressive tumor subtype.

### 3.2 MCM10‐rs2274110[A] variant stables MCM10 protein expression by increasing its SUMOylation level

**FIGURE 2:**
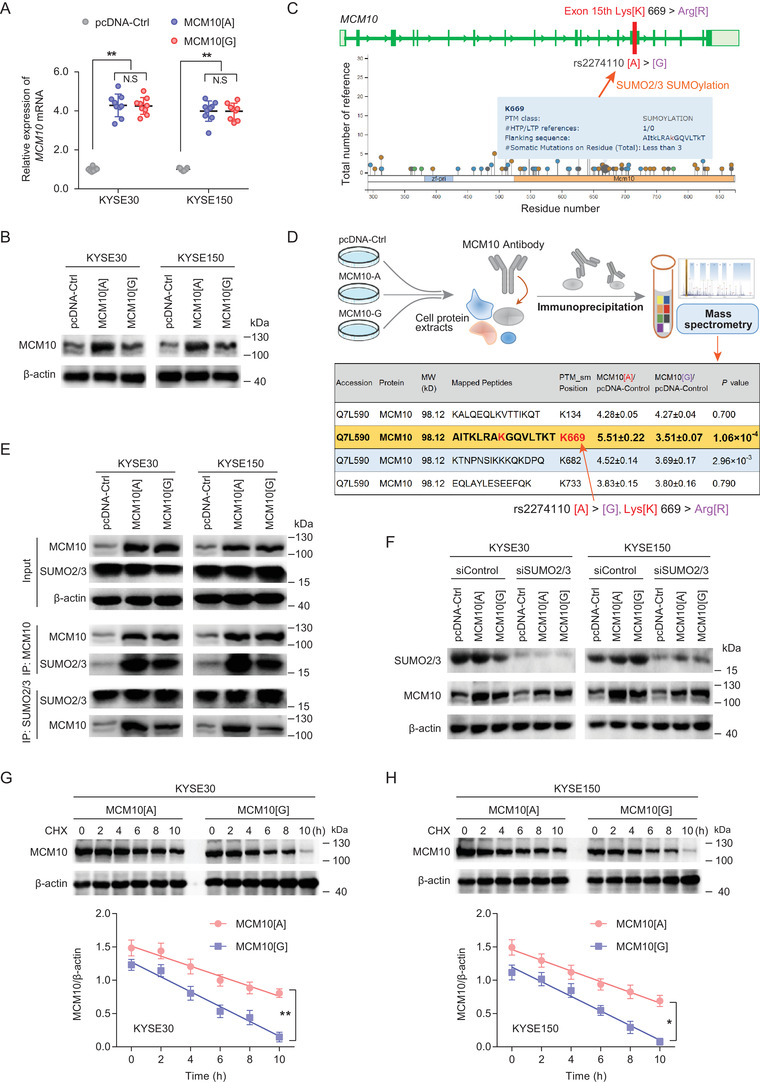
MCM10‐rs2274110[A] variant stables the expression of MCM10 protein by increasing its SUMOylation level. (A and B) The mRNA (A) and protein expression (B) levels of MCM10 were detected by qRT–PCR and western‐blotting assays, respectively, in KYSE30 and KYSE150 cells. Cells were seeded in six‐well plates after transfection with the pcDNA‐Ctrl, MCM10[A], and MCM10[G]. All qRT‐PCR data are presented as the mean ± SD from three repeated experiments, each with triplicates. **p < 0.01 were calculated using a two‐sided Student's *t*‐test. (C) Post‐translational modifications prediction of variant rs2274110 using the PhosphoSitePlus database (https://www.phosphosite.org/homeAction) revealing that rs2274110[A] > [G] causes a Lys[K]699Arg[R] change and disrupt a SUMOylation site at K669 of MCM10. (D) Proteomic screening by mass spectrometry (MS) for the identification of SUMOylation site of MCM10. *p* < 0.05 was selected as the threshold of significance. (E) Co‐IP analysis using anti‐SUMO2/3 or anti‐MCM10 antibodies. Cells were seeded in six‐well plates after transfection with the pcDNA‐Ctrl, MCM10[A] and MCM10[G]. (F) Western blotting analysis revealing the effect of SUMO2/3 on MCM10 protein expression. Cells were seeded in six‐well plates after transfection with the siRNAs targeting SUMO2/3 or siControl. Then, cells were transfected with MCM10[A], MCM10[G], or the control vector. (G and H) The degradation of MCM10 was detected by western blotting (Upper) and quantitative analysis in the presence of CHX (Below). MCM10[A] or MCM10[G] was transfected into KYSE30 (G) and KYSE150 (H) cells treated with translation inhibitor Cycloheximide (CHX, 100 μg/ml) for 0, 2, 4, 6, 8, and 10 h. **p < 0.01 values were calculated using a two‐sided Student's *t*‐test

Considering that variant rs2274110 is located at the 15th exon of MCM10, we speculated that it might have a regulatory effect on gene expression or post‐translational modifications (PTMs) of protein. When we overexpressed the same amount of transcript with MCM10‐rs2274110[A] allele or MCM10‐rs2274110[G] allele in KYSE30 and KYSE150 cell lines carrying rs2274110‐AA genotype, respectively, there are no significant differences in mRNA level of *MCM10* between both transcripts (Figure 2A). However, the protein level of MCM10 is higher in group with MCM10‐rs2274110[A] allele overexpression, compared with the group with MCM10‐rs2274110[G] allele overexpression (Figure 2B). In addition, we also measured the expression of MCM10 protein in ESCC samples with different rs2274110 genotypes and observed that the rs2274110‐AA genotype carriers have a higher MCM10 protein expression, compared to the rs2274110‐AG genotype carriers (Figure S2D). Interestingly, we further found that this variant in the *MCM10* can cause a Lys[K]669Arg[R] change and disrupt a SUMOylation site at K669 of MCM10 protein (Figure 2C). Through a mass spectrometry assay followed by overexpression of transcripts with different MCM10‐rs2274110 alleles, we further observed that the SUMOylation level of MCM10 is higher when MCM10‐[A] allele overexpression, compared with that of MCM10[G] allele overexpression (Figure 2D). Additionally, the Co‐IP assay also showed that the binding of SUMO2/3 is more enriched with the MCM10[A], compared with the MCM10[G] (Figure 2E). Interestingly, when knocking down SUMO2/3 with siRNAs, the differences of MCM10 protein levels between MCM10[A] allele and MCM10[G] allele are substantially attenuated in KYSE30 and KYSE150 cells (Figure 2F). Furthermore, we observed that the degradation rate of MCM10 protein is also shown to be lower for the MCM10[A] group, compared with the MCM10[G] group, in both ESCC cells (Figures 2G and 2H). Collectively, these results indicate that the MCM10‐rs2274110[A] allele can increase its SUMOylation level to inhibit the degradation of MCM10 protein.

### 3.3 The high expression of MCM10 confers an inferior survival of ESCC patients

**FIGURE 3:**
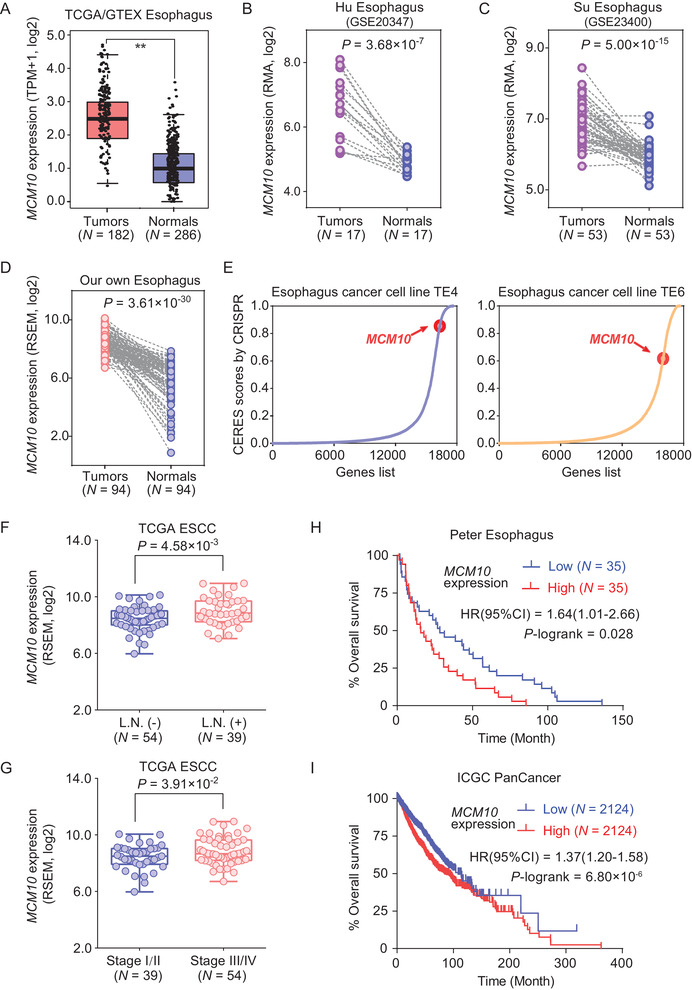
The association of MCM10 with clinical prognosis of ESCC patients. (A‐D) The expression of MCM10 is significantly upregulated in ESCC tumor tissues compared with their normal tissues from multiple cohorts including TCGA/GTEX (A), Hu (GSE20347) (B), Su (GSE23400) (C), and our own ESCC tissues (D). Data were shown as the mean ± SD, and all **p < 0.01 values were calculated by a two‐sided Student's *t*‐test in TCGA/GTEX ESCC tissues, but with a paired two‐sided Student's *t*‐test in Hu (GSE20347), Su (GSE23400), and our own ESCC tissues. (E) MCM10 is essential for cell growth with higher CERES scores in ESCA TE4 and TE6 cell lines from the data of genome‐wide CRISPR/Cas9‐based loss‐of‐function screen. Higher CERES scores demonstrate an elevated dependency of cell viability on given genes. (F and G) MCM10 expression levels were measured in ESCCs with or without lymph node (L.N.) metastasis (F) or different tumor stages (G) of ESCC from TCGA database. Data were presented as the mean ± SD, and *p* values were calculated by using a two‐sided Student's *t*‐test. (H and I) Kaplan‐Meier estimates of survival time for individuals with ESCC stratified by MCM10 expression from Peter esophagus (GSE19417), and ICGC database. *p* value and HR (95% CI) were calculated by the log‐rank test Abbreviation: ICGC, International Cancer Genome Consortium cohort.

We further evaluated whether the overexpression of *MCM10* is also associated with the clinical prognosis of ESCC patients. In contrast to adjacent normal tissues, the expression of *MCM10* was shown to be increased in tumor tissues of ESCC from four independent cohorts, including TCGA/GTEX, GEO, and our own ESCC samples, respectively (Figures 3A‐3D). Moreover, *MCM10* expression was presented extensively to be upregulated level in tumor tissues of other cancer types, and the amplification of *MCM10* also frequently occurred across multiple cancer types, including ESCC, from the TCGA database (Figures S3A and S3B). Also, we found that *MCM10* presented a high dependence degree in two different esophagus cancer cell line TE4 and TE6 based on the loss‐of‐function screens data of CRISPR/Cas9 technology^31^ (Figure 3E), suggesting the MCM10 is essential for ESCC cells proliferation. Furthermore, the high expression of *MCM10* is remarkably associated with multiple clinical prognosis traits of ESCC, such as the lymph node (L.N.) metastasis, advanced tumor stage, and poor survival (Figures 3F‐3I). In addition, in consistent with results in ESCC, the significant associations between high expression of MCM10 and the poor survival outcomes were observed across multiple cancer types (Figure S4). Collectively, these results demonstrate that the *MCM10* may not only be a prognostic marker but also play an important role in promoting ESCC progression.

### 3.4 Upregulation of MCM10 deteriorates the progression and metastasis of ESCC cells

**FIGURE 4:**
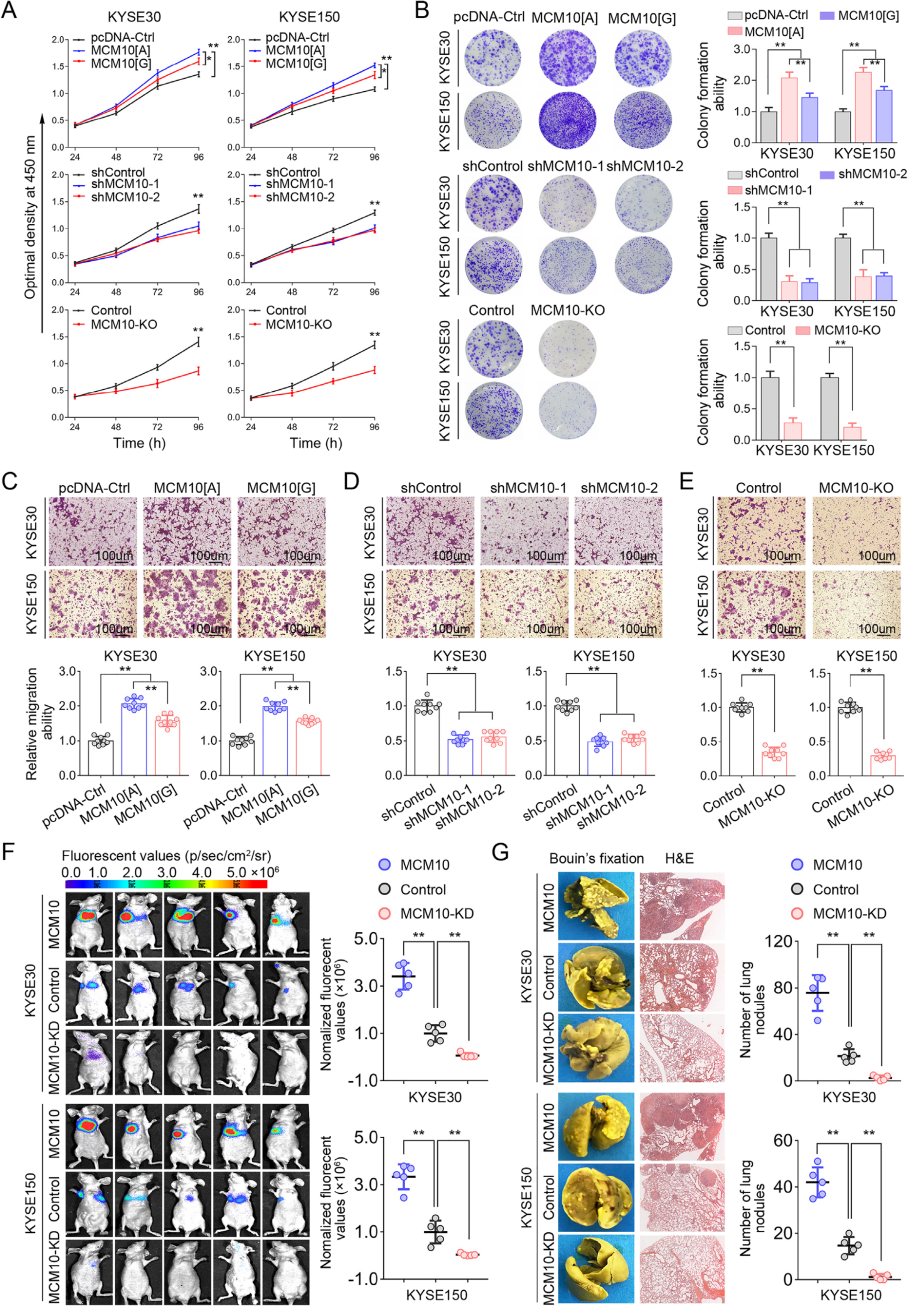
Upregulation of MCM10 promotes the proliferation and metastasis of ESCC cells. (A) The effect of MCM10[A] and MCM10[G] overexpression, MCM10 knockdown or MCM10 knockout on the proliferation rate of KYSE30 and KYSE150 cells. Data were shown as the mean ± s.e.m. from three experiments, each with six replicates. All *p < 0.05, **p < 0.01, compared with controls by a two‐sided Student's *t*‐test. (B‐E) The effect of MCM10[A] and MCM10[G] overexpression, MCM10 knockdown or MCM10 knockout on the colony formation ability (B) and the migration ability (C‐E) of ESCC cells. Results were shown as the mean ± SD from three repeated experiments and each with triplicates. **p < 0.01 was calculated using a two‐sided Student's *t*‐test. (F and G) A mouse model of lung metastases was established by tail vein injection of the indicated MCM10 overexpression or knockdown. Representative bioluminescence images (F, left) and bioluminescence signals acquired (F, right), at 8 weeks after injection, representative H&E staining of lung tissues (G, left) and the number of tumor foci on the lung surface (G, right) from the different groups are shown. Results were shown as the means ± SD for five mice in per group. All **p < 0.01, compared with control cells by a two‐sided Student's *t*‐test

To further illuminate the functional role of *MCM10* in the progression of ESCC, we tested the ESCC cell malignant phenotypes upon the knockdown, knockout of MCM10, or overexpression of transcripts carrying different MCM10‐rs2274110 allele, respectively. We observed that the overexpression of MCM10[A] allele can prominently enhance the proliferation ability of both ESCC cells, in contrast to the overexpression of MCM10[G] allele or the control vector (Figure 4A). While both MCM10 knockdown by siRNAs and MCM10 knockout by CRISPR/Cas9 significantly inhibited the proliferation rate of both ESCC cells (Figure 4A). Consistently, the results present similar trends in the colony formation assays (Figure 4B). Similarly, in the trans‐well assays, the ESCC cells also showed higher migration ability in group with overexpression of MCM10[A] allele, compared to the MCM10[G] allele or the control vector group (Figure 4C). Expectedly, we also found that the knockdown or knockout of MCM10 can significantly attenuate the progression ability of ESCC cells (Figures 4D and 4E). Furthermore, we also found that the MCM10 promotes ESCC metastasis *in vivo*. The lung metastasis model revealed that the stable overexpression of MCM10 in both KYSE30 and KYSE150 cells can significantly facilitate colonization of the lung and increases the number of lung metastases, whereas the knockdown of MCM10 in both ESCC cells substantially inhibited these effects (Figures 4F and 4G). These findings in *vitro* and in *vivo* collectively reveal that the upregulation of MCM10 substantially enhances ESCC cells growth and migration.

### 3.5 Upregulation of MCM10 induces the genomic instability of ESCC cells

**FIGURE 5:**
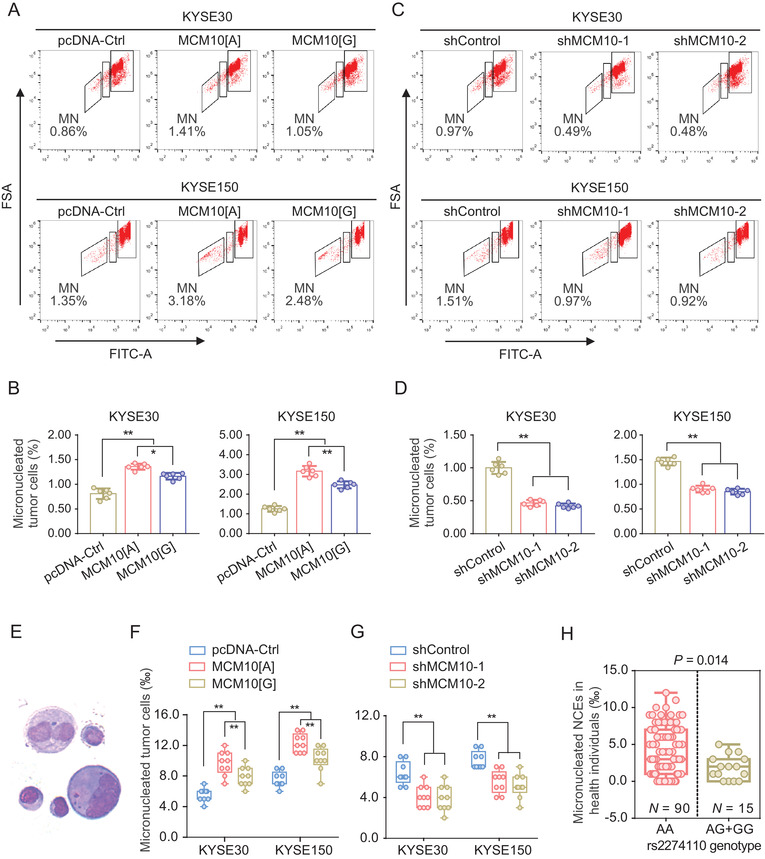
Upregulation of MCM10 induces the genomic instability of ESCC cells. (A‐D) The effect of MCM10[A] and MCM10[G] overexpression (A and B) or MCM10 knockdown (C and D) on MN frequency in KYSE30 and KYSE150 cells by flow cytometric analysis. Flow cytometry images (A and C) and quantitative statistics (B and D) were shown as the means ± SD from a representative result of three repeated experiments and each with six replicates. **p < 0.01 was calculated by a two‐sided Student's *t*‐test. (E‐G) The effect of MCM10[A] and MCM10[G] overexpression (F) or MCM10 knockdown (G) on MN frequency in KYSE30 and KYSE150 cells by cytokinesis block (CB) micronucleus testing. Representative images (E) and quantitative statistics (F and G) were shown as the as the medians (min to max) from three repeated experiments, each with triplicates. **p < 0.01 was calculated by a two‐sided Student's *t*‐test. (H) Carries with the MCM10‐rs2274110 AA genotype had higher MN frequency in 105 healthy individuals. Results were shown as the medians (min to max), and *p* value was calculated using a two‐sided Student's *t*‐test

Previous researches showed that the deregulation of CDT1 or depletion of its inhibitor geminin may lead to the DNA over‐replication and caused genomic instability.^17^, ^32^, ^33^ As MCM10 has synergistic effect with CDT1 in regulating replication licensing, we then tested whether the *MCM10* enhances ESCC progression by inducing the genomic instability. We interestingly observed that the proportion of micronucleated KYSE30 and KYSE150 cells are significantly increased in the group with the overexpression of MCM10[A] allele, compared with the group with overexpression of the MCM10[G] allele or the control vector (Figures 5A and 5B). When MCM10 was successfully knocked down, we found a significantly decreasing in the proportion of both the micronucleated ESCC KYSE30 and KYSE150 cells (Figures 5C and5 D). Expectedly, these results are consistent with the results measured with another micronucleus testing method (Figures 5E‐5G). In addition, we further measured the proportion of micronucleated lymphocytes in 105 healthy subjects carrying different rs2274110 genotypes and found that subjects with AA genotype have a more amount of micronucleated lymphocytes, compared the subjects carrying AG/GG genotypes (Figure 5H). Collectively, these results demonstrate that aberrant upregulation of MCM10 may facilitate the progression of ESCC through inducing the genomic instability.

### 3.6 The MCM10 inhibitors Suramin and its analogues may serve as potential anti‐cancer agents for ESCC

**FIGURE 6:**
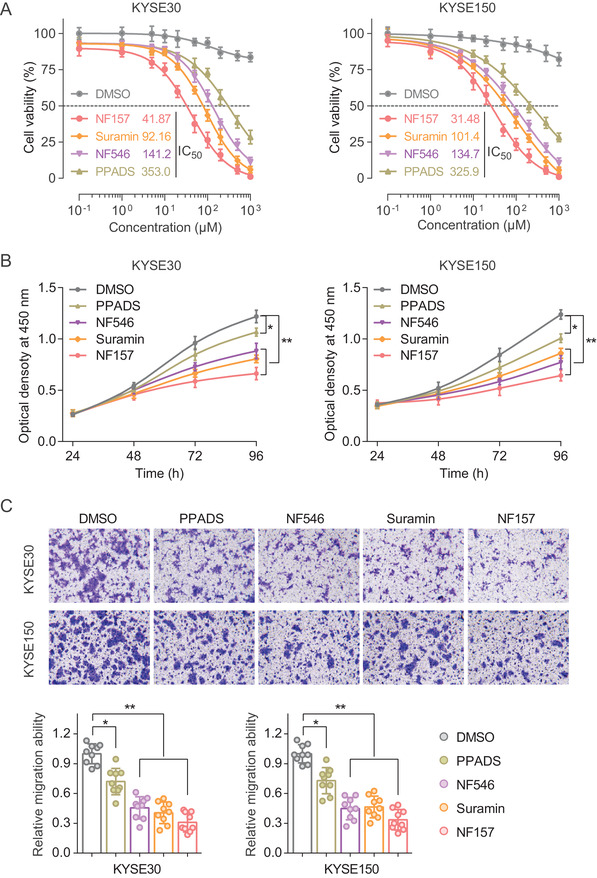
The MCM10 inhibitors Suramin and its analogues inhibit cell proliferation and migration of ESCC cells. (A) Dose–response curves of KYSE30 and KYSE150 cell lines treated with Suramin and its analogues (NF157, NF546, and PPADS) with an endpoint measurement at 48 h. Data were shown as the means ± SD from three repeated experiments, each with triplicates. IC50, half‐maximal inhibitory concentration. (B) The effect of the Suramin and its analogues on the proliferation rate of ESCC cell lines. Results were shown as the mean ± s.e.m. from three experiments, each with six replicates. *p < 0.05, **p < 0.01, compared with controls by a two‐sided Student's *t*‐test. (C) The effect of Suramin and its analogues on cell migration ability of ESCC cells. Results were shown as the mean ± SD from three repeated experiments, each with triplicates. **p < 0.01 was calculated using a two‐sided Student's *t*‐test

Furthermore, we aimed to evaluate whether previously reported MCM10 inhibitors Suramin and its analogues (NF157, NF546, and PPADS)^29^, ^30^ could inhibit ESCC cells growth and metastasis. The results revealed that these molecule inhibitors treatments can effectively inhibit the cell viability of both KYSE30 and KYSE150 cells, and presented in a dose dependent manner (Figure 6A). Moreover, the cell proliferation and migration abilities of both KYSE30 and KYSE150 cells were significantly suppressed under these drugs treatment (Figures 6B and 6C). Interestingly, in these several drugs, the NF157 had the most effective and resulted in a 69.03% or 66.41% decrease in the migration ability of KYSE30 or KYSE150 cells, respectively. Collectively, these findings indicate that the MCM10 inhibitors Suramin and its analogues can effectively suppress the oncogenic transformation induced by ESCC cells.

## 4 DISCUSSION

Through comprehensively integrating two‐stage population survival analysis and multipronged biochemical experiments in this study, we identified a germline variant rs2274110 in *MCM10* that confers an inferior survival of ESCC patients. This functional variant can upregulate SUMOylation level of MCM10 protein to result in its protein aberrant overexpression, which further substantially facilitate ESCC progression and metastasis in vitro and in vivo probably by fueling DNA over‐replication and genomic instability. Importantly, MCM10 is shown to be served as a promising prognostic biomarker and MCM10 inhibitors Suramin, and its analogues are revealed to present potential anti‐cancer effects for ESCC.

Reversible PTMs of protein have been well documented as important biological mechanisms that dynamically and rapidly response to extracellular and intracellular stimulation and influence evolutionary development and progression of diseases.^34^ SUMOylation is one of PTMs and revealed to be closely involved in various physiological processes of mammalian cells, such as signal transduction, genome stability, the DNA damage responses and protein trafficking,^20^ and one that seems to be upregulated in many diseases.^35^ Interestingly, in contrast to ubiquitylation of protein, SUMOylation does not degrade target proteins, while seems to modulate their subcellular compartmentalization and reinforce their stability.^36^ Additionally, Bao et al also revealed that the K501 SUMOylation on BACE1 protein enhances its stability and escalates the protease activity in Alzheimer's disease.^36^ In consistent with these findings, we also found that the rs2274110[A] variant stables MCM10 protein expression by increasing its SUMOylation level and presents a shorter survival outcome for ESCC. These findings underline the clinical potential and importance of targeting the SUMOylation machinery for disease therapy.

Replication licensing is a well‐organized process to assure the proper duplication and assembly of genome during cell cycle progression. Recently, it was suggested that both the reduction and the ectopic or increasing of origin licensing could result in replication stress and re‐replicated process of DNA, thus resulting in occurrence of genomic instability.^37^, ^38^ Especially, in cancer cells, most of the replication licensing factors were upregulated, and the DNA re‐replication (over‐replication) was induced.^33^ The DNA re‐replication leads to the accumulation of single‐strand DNA (ssDNA) and the formation of DNA double‐strand breaks (DSBs), which are sources of genomic instability.^39^, ^40^

The MCM family genes are essential factors in eukaryotic genome replication initiation and elongation by activating and stabling the C45‐MCM2‐7‐GINS (CMG) complex and leading to DNA unwinding and replication fork assembly.^41^, ^42^, ^43^ It was reported that in normal cells of eukaryotes, loss or mutations in the MCMs are associated with genomic instability, possibly due to a failure to license additional replication origins following a replication fork failure.^10^ However, in tumor cells, depletion of the MCMs, for example MCM7, was found to suppress the tumor growth and progression of non‐small cell lung cancer and glioblastoma in *vitro* and in *vivo*.^14^, ^15^ As for MCM10, it frequently presents overexpression and amplification in multiple cancer types and contributes to poor prognosis.^44^, ^45^, ^46^ Moreover, the MCM10 expression is activated by some oncogenes, such as N‐MYC and Ewing's sarcoma‐derived oncogenes in neuroblastoma and Ewing's tumors.^47^ Intriguingly, MCM10 overexpression in yeast had also been reported to drive genome instability.^48^ In consistent with these finding, here our study also found that *MCM10* is overexpressed in tumor tissues of ESCC and prominently correlated with the inferior survival of ESCC patients. The aberrant overexpression of *MCM10* was shown to significantly provoke ESCC cells proliferation and migration abilities and fuel more micronucleated ESCC cells. These observations suggested at least two hypotheses for the function of MCM10 in ESCC: First, the upregulation of MCM10 may simply be one of replication licensing factors in response to the rapid increasing rate of proliferation in tumor cells which need MCM10 to overcome limitations for DNA replication dictated by active cell replication and altered cell cycle control. Second, aberrant overexpression of MCM10 might act as an augmenting force in cancer development or progression to drive over‐replication and genomic instability. Considering that aberrant overexpression of MCM10 in ESCC tumor cells might induce DNA over‐replication, moderately decreasing expression of MCM10 might be helpful for suppressing the proliferation and migration of ESCC cells. Noticeably, whether MCM10 plays a causal role in cancer cells proliferation, migration or genome instability is an interesting issue, which should be further interrogated.

*MCM10*, as a replication licensing factor, appears to be a promising anticancer drug target. Previous researches support this idea revealing that in contrast to normal cells, the ablation of replication licensing factors can effectively reduce replication stress specifically in cancer cells.^49^, ^50^ In addition, a high‐throughput cell‐based screening for licensing inhibitors also prioritized a small‐molecule inhibitor for replication licensing factor, RL5a, which can restrain the binding of ORC to DNA and compromise MCM2–7 loading onto origins, thereby successfully reducing the proliferation of cancer cells.^51^ Moreover, licensing inhibitors might also be shown to work synergistically with existing chemotherapeutic drugs. These findings pave the way for replication licensing factors serving as an attractive anti‐cancer target. In our study, we also indicated that the MCM10 inhibitors Suramin and its analogues (NF157, NF546, and PPADS) can block both cancer cells proliferation and migration in the ESCC. Besides, previous researches have also revealed that Suramin can effectively inhibit growth of tumor xenografts in nude mice, such as osteosarcoma, breast cancer, and Ewing's sarcoma^52^, ^53^, ^54^ and moderately extend the lifespan of animals with breast cancer lung metastasis.^55^ Additionally, suramin has also been demonstrated to enhancer paclitaxel activity against tumor growth or metastasis of both lung cancer and breast cancer in vivo.^56^, ^57^ These findings provide evidence suggesting that MCM10 small‐molecule inhibitors may serve as potential anti‐cancer agents in treating advanced cancer. However, it should be noted that Suramin and its analogues exert a wide variety of biological effects and that are not specific to MCM10 alone. They have also been found to inhibit other proteins involved in other tumor signaling pathways, such as DNA polymerases, Wnt, and insulin‐like growth factor‐I receptor.^53^, ^54^, ^58^ A phase I/II clinical trial related to suramin therapy in hormone refractory prostate cancer patients also uncovered that suramin had a neurologic toxicity, although antitumor activity was observed.^59^ Further investigations are warranted to deconstruct the precise mechanisms and effective targets of this drug before its clinical use. In addition, considering that MCM10 inhibitors Suramin and its analogues (NF157, NF546, and PPADS) are not specific to MCM10 alone, researchers in many disciplines are encouraged to find and deconstruct new specific drugs in the future.

In summary, through a two‐stage survival analysis for 1407 ESCC patients, we identified a replication licensing factor germline variant, which might be ideal as a potential prognostic biomarker stratifying ESCC patients effectively. Mechanistically, we further demonstrated that replication licensing factor MCM10 can deteriorate the progression and metastasis of ESCC and induce DNA over‐replication and genomic instability. These findings collectively indicate that preexisting hereditary genetics of individuals can effectively influence progression and prognosis outcomes of ESCC and provide more sights into the personalized therapy for cancer.

## DECLARATIONS

## ETHICS APPROVAL AND CONSENT TO PARTICIPATE

Informed consent was obtained from all participants, and this study was approved by the institutional review board of HUST ([2016]IEC(S160)). All animal experimental procedures were performed in accordance with the relevant institutional and national guidelines and approved by the Institutional Animal Care and Use Committee of HUST [IACUC_Number: S2344].

## CONSENT FOR PUBLICATION

Consent to publish has been obtained from all authors.

## CONFLICT OF INTEREST

The authors report no conflict of interest.

## AUTHOR CONTRIBUTIONS

Xiaoping Miao and Jiang Chang were the overall principal investigators in this study who conceived the study and obtained financial support, were responsible for the study design and supervised the entire study. Jianbo Tian and Jiang Chang performed statistical analyses, interpreted the data, and drafted the initial manuscript. Jianbo Tian, Zequn Lu, Siyuan Niu, Shanshan Zhang, Pingting Ying, Lu Wang, Ming Zhang, Yimin Cai, Tianyi Dong, and Ying Zhu performed laboratory experiments. Rong Zhong, Zhihua Wang, and Xiaoping Miao were responsible for patient recruitment and sample preparation. All authors approved the final report for publication.

## Supporting information

Supporting information

## Data Availability

The MCM10 mRNA expression data were derived from the TCGA Research Network (http://cancergenome.nih.gov/), GETX (https://commonfund.nih.gov/GTEx/), and GEO database (https://www.ncbi.nlm.nih.gov/geo/). Other data associated with this study are present in the paper and Supplementary information or are available from the corresponding author upon reasonable request.

## References

[1] Chen W , Zheng R , Baade PD , et al. Cancer statistics in China, 2015. CA Cancer J. Clin.. 2016;66(2):115–132. 26808342 10.3322/caac.21338

[2] Lagergren J , Smyth E , Cunningham D , Lagergren P . Oesophageal cancer. Lancet (London, England). 2017;390(10110):2383–2396. 10.1016/S0140-6736(17)31462-9 28648400

[3] Guo YM , Chen JR , Feng YC , et al. Germline polymorphisms and length of survival of nasopharyngeal carcinoma: an exome‐wide association study in multiple cohorts. Advanced science (Weinheim, Baden‐Wurttemberg, Germany). 2020;7(10):1903727. 10.1002/advs.201903727 PMC7237860 32440486

[4] Wu C , Li D , Jia W , et al. Genome‐wide association study identifies common variants in SLC39A6 associated with length of survival in esophageal squamous‐cell carcinoma. Nat Genet. 2013;45(6):632–638. 23644492 10.1038/ng.2638

[5] Bakhoum SF , Ngo B , Laughney AM , et al. Chromosomal instability drives metastasis through a cytosolic DNA response. Nature. 2018;553(7689):467–472. 29342134 10.1038/nature25432 PMC5785464

[6] Bakhoum SF , Cantley LC . The multifaceted role of chromosomal instability in cancer and its microenvironment. Cell. 2018;174(6):1347–1360. 30193109 10.1016/j.cell.2018.08.027 PMC6136429

[7] Fragkos M , Ganier O , Coulombe P , Mechali M . DNA replication origin activation in space and time. Nat. Rev. Mol. Cell Biol.. 2015;16(6):360–374. 25999062 10.1038/nrm4002

[8] Blow JJ , Gillespie PJ . Replication licensing and cancer–a fatal entanglement?. Nat. Rev. Cancer. 2008;8(10):799–806. 18756287 10.1038/nrc2500 PMC2577763

[9] Ibarra A , Schwob E , Mendez J . Excess MCM proteins protect human cells from replicative stress by licensing backup origins of replication. Proc Natl Acad Sci U S A. 2008;105(26):8956–8961. 18579778 10.1073/pnas.0803978105 PMC2449346

[10] Ge XQ , Jackson DA , Blow JJ . Dormant origins licensed by excess Mcm2‐7 are required for human cells to survive replicative stress. Genes Dev.. 2007;21(24):3331–3341. 18079179 10.1101/gad.457807 PMC2113033

[11] Ramnath N , Hernandez FJ , Tan DF , et al. MCM2 is an independent predictor of survival in patients with non‐small‐cell lung cancer. J Clin Oncol. 2001;19(22):4259–4266. 11709570 10.1200/JCO.2001.19.22.4259

[12] Kwok HF , Zhang SD , McCrudden CM , et al. Prognostic significance of minichromosome maintenance proteins in breast cancer. American journal of cancer research. 2015;5(1):52–71. 25628920 PMC4300722

[13] Ha SA , Shin SM , Namkoong H , et al. Cancer‐associated expression of minichromosome maintenance 3 gene in several human cancers and its involvement in tumorigenesis. Clinical cancer research : an official journal of the American Association for Cancer Research. 2004;10(24):8386–8395. 15623617 10.1158/1078-0432.CCR-04-1029

[14] Toyokawa G , Masuda K , Daigo Y , et al. Minichromosome maintenance protein 7 is a potential therapeutic target in human cancer and a novel prognostic marker of non‐small cell lung cancer. Mol. Cancer. 2011;10:65. 21619671 10.1186/1476-4598-10-65 PMC3125391

[15] Erkan EP , Strobel T , Lewandrowski G , et al. Depletion of minichromosome maintenance protein 7 inhibits glioblastoma multiforme tumor growth in vivo. Oncogene. 2014;33(39):4778–4785. 24166506 10.1038/onc.2013.423

[16] Liontos M , Koutsami M , Sideridou M , et al. Deregulated overexpression of hCdt1 and hCdc6 promotes malignant behavior. Cancer Res.. 2007;67(22):10899–10909. 18006835 10.1158/0008-5472.CAN-07-2837

[17] Munoz S , Bua S , Rodriguez‐Acebes S , et al. In vivo DNA re‐replication elicits lethal tissue dysplasias. Cell Rep.. 2017;19(5):928–938. 28467906 10.1016/j.celrep.2017.04.032

[18] Blow JJ , Dutta A . Preventing re‐replication of chromosomal DNA. Nat. Rev. Mol. Cell Biol.. 2005;6(6):476–486. 15928711 10.1038/nrm1663 PMC2688777

[19] Petropoulos M , Champeris Tsaniras S , Taraviras S , Lygerou Z . Replication licensing aberrations, replication stress, and genomic instability. Trends Biochem. Sci.. 2019;44(9):752–764. 31054805 10.1016/j.tibs.2019.03.011

[20] Seeler JS , Dejean A . SUMO and the robustness of cancer. Nat. Rev. Cancer. 2017;17(3):184–197. 28134258 10.1038/nrc.2016.143

[21] Flotho A , Melchior F . Sumoylation: a regulatory protein modification in health and disease. Annu. Rev. Biochem.. 2013;82:357–385. 23746258 10.1146/annurev-biochem-061909-093311

[22] Hanahan D , Weinberg RA . Hallmarks of cancer: the next generation. Cell. 2011;144(5):646–674. 21376230 10.1016/j.cell.2011.02.013

[23] Chang J , Zhong R , Tian J , et al. Exome‐wide analyses identify low‐frequency variant in CYP26B1 and additional coding variants associated with esophageal squamous cell carcinoma. Nat Genet. 2018;50(3):338–343. 29379198 10.1038/s41588-018-0045-8

[24] Kulak NA , Pichler G , Paron I , Nagaraj N , Mann M . Minimal, encapsulated proteomic‐sample processing applied to copy‐number estimation in eukaryotic cells. Nat. Methods. 2014;11(3):319–324. 24487582 10.1038/nmeth.2834

[25] Andrews GL , Simons BL , Young JB , Hawkridge AM , Muddiman DC . Performance characteristics of a new hybrid quadrupole time‐of‐flight tandem mass spectrometer (TripleTOF 5600). Anal. Chem.. 2011;83(13):5442–5446. 21619048 10.1021/ac200812d PMC3138073

[26] Tian J , Zhu Y , Rao M , et al. N‐methyladenosine mRNA methylation of regulates AKT signalling to promote PTEN‐deficient pancreatic cancer progression. Gut. 2020;69(12):2180–2192. 32312789 10.1136/gutjnl-2019-320179

[27] UniProt Consortium T . UniProt: the universal protein knowledgebase. Nucleic Acids Res.. 2018;46(5):2699. 29425356 10.1093/nar/gky092 PMC5861450

[28] Fenech M . The cytokinesis‐block micronucleus technique: a detailed description of the method and its application to genotoxicity studies in human populations. Mutat. Res.. 1993;285(1):35–44. 7678131 10.1016/0027-5107(93)90049-l

[29] Paulson CN , John K , Baxley RM , et al. The anti‐parasitic agent suramin and several of its analogues are inhibitors of the DNA binding protein Mcm10. Open biology. 2019;9(8):190117. 31409229 10.1098/rsob.190117 PMC6731595

[30] Jindal HK , Anderson CW , Davis RG , Vishwanatha JK . Suramin affects DNA synthesis in HeLa cells by inhibition of DNA polymerases. Cancer Res.. 1990;50(24):7754–7757. 2174730

[31] Meyers RM , Bryan JG , McFarland JM , et al. Computational correction of copy number effect improves specificity of CRISPR‐Cas9 essentiality screens in cancer cells. Nat. Genet.. 2017;49(12):1779–1784. 29083409 10.1038/ng.3984 PMC5709193

[32] Klotz‐Noack K , McIntosh D , Schurch N , Pratt N , Blow JJ . Re‐replication induced by geminin depletion occurs from G2 and is enhanced by checkpoint activation. J. Cell Sci.. 2012;125(Pt 10):2436–2445. 22366459 10.1242/jcs.100883 PMC3481538

[33] Hernandez‐Carralero E , Cabrera E , Alonso‐de Vega I , Hernandez‐Perez S , Smits VAJ , Freire R . Control of DNA replication initiation by ubiquitin. Cells. 2018;7(10):146. 10.3390/cells7100146 PMC6211026 30241373

[34] Flotho A , Melchior F . Sumoylation: a regulatory protein modification in health and disease. Annu. Rev. Biochem.. 2013;82:357–385. 23746258 10.1146/annurev-biochem-061909-093311

[35] Eifler K , Vertegaal ACO . SUMOylation‐mediated regulation of cell cycle progression and cancer. Trends Biochem. Sci.. 2015;40(12):779–793. 26601932 10.1016/j.tibs.2015.09.006 PMC4874464

[36] Bao J , Qin M , Mahaman YAR , et al. BACE1 SUMOylation increases its stability and escalates the protease activity in Alzheimer's disease. Proc. Natl. Acad. Sci. USA. 2018;115(15):3954–3959 29581300 10.1073/pnas.1800498115 PMC5899489

[37] Gonzalez MA , Tachibana KE , Laskey RA , Coleman N . Control of DNA replication and its potential clinical exploitation. Nat. Rev. Cancer. 2005;5(2):135–141. 15660109 10.1038/nrc1548

[38] Gaillard H , Garcia‐Muse T , Aguilera A . Replication stress and cancer. Nat. Rev. Cancer. 2015;15(5):276–289. 25907220 10.1038/nrc3916

[39] Davidson IF , Li A , Blow JJ . Deregulated replication licensing causes DNA fragmentation consistent with head‐to‐tail fork collision. Mol. Cell. 2006;24(3):433–443. 17081992 10.1016/j.molcel.2006.09.010 PMC1819398

[40] Liu E , Lee AY , Chiba T , Olson E , Sun P , Wu X . The ATR‐mediated S phase checkpoint prevents rereplication in mammalian cells when licensing control is disrupted. J. Cell Biol.. 2007;179(4):643–657. 18025301 10.1083/jcb.200704138 PMC2080923

[41] Yeeles JT , Deegan TD , Janska A , Early A , Diffley JF . Regulated eukaryotic DNA replication origin firing with purified proteins. Nature. 2015;519(7544):431–435. 25739503 10.1038/nature14285 PMC4874468

[42] Thu YM , Bielinsky AK . MCM10: one tool for all‐Integrity, maintenance and damage control. Semin. Cell Dev. Biol.. 2014;30:121–130. 24662891 10.1016/j.semcdb.2014.03.017 PMC4043890

[43] van Deursen F , Sengupta S , De Piccoli G , Sanchez‐Diaz A , Labib K . Mcm10 associates with the loaded DNA helicase at replication origins and defines a novel step in its activation. EMBO J.. 2012;31(9):2195–2206. 22433841 10.1038/emboj.2012.69 PMC3343467

[44] Das M , Prasad SB , Yadav SS , et al. Over expression of minichromosome maintenance genes is clinically correlated to cervical carcinogenesis. PLoS One. 2013;8(7):e69607. 23874974 10.1371/journal.pone.0069607 PMC3714251

[45] Cui F , Hu J , Ning S , Tan J , Tang H . Overexpression of MCM10 promotes cell proliferation and predicts poor prognosis in prostate cancer. Prostate. 2018;78(16):1299–1310. 30095171 10.1002/pros.23703 PMC6282949

[46] Mahadevappa R , Neves H , Yuen SM , et al. DNA replication licensing protein MCM10 promotes tumor progression and is a novel prognostic biomarker and potential therapeutic target in breast cancer. Cancers. 2018;10(9):282. 10.3390/cancers10090282 PMC6162382 30135378

[47] Garcia‐Aragoncillo E , Carrillo J , Lalli E , et al. DAX1, a direct target of EWS/FLI1 oncoprotein, is a principal regulator of cell‐cycle progression in Ewing's tumor cells. Oncogene. 2008;27(46):6034–6043. 18591936 10.1038/onc.2008.203

[48] Haworth J , Alver RC , Anderson M , Bielinsky A‐K . Ubc4 and Not4 regulate steady‐state levels of DNA polymerase‐α to promote efficient and accurate DNA replication. Mol Biol Cell. 2010;21(18):3205–3219. 20660159 10.1091/mbc.E09-06-0452 PMC2938386

[49] McIntosh D , Blow JJ . Dormant origins, the licensing checkpoint, and the response to replicative stresses. Cold Spring Harb. Perspect. Biol.. 2012;4(10):a012955. 22904560 10.1101/cshperspect.a012955 PMC3475168

[50] Zimmerman KM , Jones RM , Petermann E , Jeggo PA . Diminished origin‐licensing capacity specifically sensitizes tumor cells to replication stress. Molecular cancer research : MCR. 2013;11(4):370–380. 23364533 10.1158/1541-7786.MCR-12-0491 PMC3797919

[51] Gardner NJ , Gillespie PJ , Carrington JT , et al. The high‐affinity interaction between ORC and DNA that is required for replication licensing is inhibited by 2‐arylquinolin‐4‐amines. Cell chemical biology. 2017;24(8):981–992. 28781123 10.1016/j.chembiol.2017.06.019 PMC5563080

[52] Walz TM , Abdiu A , Wingren S , Smeds S , Larsson SE , Wasteson A . Suramin inhibits growth of human osteosarcoma xenografts in nude mice. Cancer Res.. 1991;51(13):3585–3589. 2054794

[53] Koval A , Ahmed K , Katanaev VL . Inhibition of Wnt signalling and breast tumour growth by the multi‐purpose drug suramin through suppression of heterotrimeric G proteins and Wnt endocytosis. Biochem. J.. 2016;473(4):371–381. 26604320 10.1042/BJ20150913

[54] Scotlandi K , Benini S , Nanni P , et al. Blockage of insulin‐like growth factor‐I receptor inhibits the growth of Ewing's sarcoma in athymic mice. Cancer Res.. 1998;58(18):4127–4131. 9751624

[55] Cheng B , Gao F , Maissy E , Xu P . Repurposing suramin for the treatment of breast cancer lung metastasis with glycol chitosan‐based nanoparticles. Acta Biomater. 2019;84:378–390. 30528604 10.1016/j.actbio.2018.12.010 PMC6362832

[56] Song S , Wientjes MG , Walsh C , Au JL . Nontoxic doses of suramin enhance activity of paclitaxel against lung metastases. Cancer Res.. 2001;61(16):6145–6150. 11507065

[57] Song S , Yu B , Wei Y , Wientjes MG , Au JLS . Low‐dose suramin enhanced paclitaxel activity in chemotherapy‐naive and paclitaxel‐pretreated human breast xenograft tumors. Clinical cancer research : an official journal of the American Association for Cancer Research. 2004;10(18 Pt 1):6058–6065. 15447990 10.1158/1078-0432.CCR-04-0595

[58] Jindal HK , Anderson CW , Davis RG , Vishwanatha JK . Suramin affects DNA synthesis in HeLa cells by inhibition of DNA polymerases. Cancer Res.. 1990;50(24):7754–7757. 2174730

[59] Bowden CJ , Figg WD , Dawson NA , et al. A phase I/II study of continuous infusion suramin in patients with hormone‐refractory prostate cancer: toxicity and response. Cancer Chemother Pharmacol. 1996;39(1–2):1–8. 8995493 10.1007/s002800050531

