# Supplementary material for: Aberrant MCM10 SUMOylation induces genomic instability mediated by a genetic variant associated with survival of esophageal squamous cell carcinoma
Source: Clin Transl Med. 2021 Jun 27;11(6):e485. doi: 10.1002/ctm2.485 (PMC8236122; doi:10.1002/ctm2.485)
Supplement: Supplementary file 1 — Supporting information [file CTM2-11-e485-s001.docx]

| **Supplementary Tables:**  **Table S1. Characteristics of individuals with ESCC in the discovery, replication and combined samples** | | | | | | | | | | | | | | | |
| --- | --- | --- | --- | --- | --- | --- | --- | --- | --- | --- | --- | --- | --- | --- | --- |
|  | | **Discovery stage (*N* = 904)** | | |  | **Replication stage (*N* = 503)** | | | |  | **Combined stages (*N* = 1407)** | | | | |
|  |  | **Patients** | **MST** | ***P*^†^** |  | **Patients** | | **MST** | ***P*^†^** |  | **Patients** | | **MST** | ***P*^†^** |  |
|  |  | **No. (%)** | **(Months)** |  |  | **No. (%)** | | **(Months)** |  |  | **No. (%)** | | **(Months)** |  |  |
| Vital status | |  |  |  |  |  | |  |  |  |  | |  |  |  |
|  | Dead | 696 (77.0) |  |  |  | 310 (61.6) | |  |  |  | 1006 (71.5) | |  |  |  |
|  | Alive | 208 (23.0) |  |  |  | 193 (38.4) | |  |  |  | 401 (28.5) | |  |  |  |
| Age at diagnosis | |  |  | 0.0540 | |  |  |  | 0.3587 | |  |  |  | 0.0993 |  |
|  | ≤ 60 years | 450 (49.8) | 31 |  |  | 240 (47.7) | | 21 |  |  | 690 (49.0) | | 28 |  |  |
|  | > 60 years | 454 (50.2) | 26 |  |  | 263 (52.3) | | 21 |  |  | 717 (51.0) | | 25 |  |  |
| Sex | |  |  | 0.3950 | |  |  |  | 0.9930 | |  |  |  | 0.0940 |  |
|  | Male | 726 (80.3) | 29 |  |  | 423 (84.1) | | 20 |  |  | 1149 (81.7) | | 25 |  |  |
|  | Female | 178 (19.7) | 31 |  |  | 80 (15.9) | | 27 |  |  | 258 (18.3) | | 30 |  |  |
| Smoking status | |  |  | 0.3654 | |  |  |  | 0.0910 | |  |  |  | 0.0875 |  |
|  | Nonsmoker | 308 (34.1) | 31 |  |  | 153 (30.4) | | 23 |  |  | 461 (32.8) | | 28 |  |  |
|  | Smoker | 596 (65.9) | 28 |  |  | 350 (69.6) | | 20 |  |  | 946 (67.2) | | 24 |  |  |
| Alcohol consumption | |  |  | 0.9014 | |  |  |  | 0.0390 | |  |  |  | 0.1796 |  |
|  | Nondrinker | 390 (43.1) | 28 |  |  | 187 (37.2) | | 24 |  |  | 577 (41.0) | | 27 |  |  |
|  | Drinker | 514 (56.9) | 30 |  |  | 316 (62.8) | | 20 |  |  | 830 (59.0) | | 25 |  |  |
| Tumor stage | |  |  | <0.0001 | |  |  |  | <0.0001 | |  |  |  | <0.0001 |  |
|  | I | 82 (9.1) | 94 |  |  | 42 (8.4) | | 30 |  |  | 124 (8.8) | | 48 |  |  |
|  | II | 352 (38.9) | 48 |  |  | 174 (34.6) | | 39 |  |  | 526 (37.4) | | 47 |  |  |
|  | III | 397 (43.9) | 20 |  |  | 239 (47.5) | | 17 |  |  | 636 (45.2) | | 19 |  |  |
|  | IV | 73 (8.1) | 10 |  |  | 48 (9.5) | | 8 |  |  | 121 (8.6) | | 9 |  |  |
| Abbreviation: MST, Median survival time.  ***^†^*** *P* values were calculated by the log-rank test. | | | | | | | | | | | | | | | |

| **Table S2. Probes or primers sequence used in the study** | | |
| --- | --- | --- |
| RT-PCR (5'-3') | | |
| *GAPDH* | Forward | C​C​T​C​C​C​G​C​T​T​C​G​C​T​C​T​C​T |
|  | Reverse | T​G​G​C​G​A​C​G​C​A​A​A​A​G​A​A​G​A​T​ |
| *MCM10* | Forward | GTCACCACCACCAAGACCAA |
|  | Reverse | TGGCAGGCTCCAATTCATCC |
| siRNAs/shRNAs | | |
| siSUMO2/3 | siRNA1 | GCATACACCACTTAGTAAA |
|  | siRNA2 | GGAGGATGAAGATACAATT |
|  | siRNA3 | AGCAGACGGGAGGTGTCTA |
| shMCM10 | shRNA1 | GGAGAAACAGGAGAGACAAGA |
|  | shRNA2 | GCAAATGAAGGCCTTACAAGA |
|  | shRNA3 | GCTCGAACACCAAAGGCTTCA |
| sgRNAs |  |  |
| MCM10 | sgRNA1 | ATAAGATTCACCGTCGCCGTCGG |
|  | sgRNA2 | AACTTCTTGACGCGGGAAAATGG |
|  | sgRNA3 | GTCTCTGCTGACCGCACTGCTGG |

| **Table S3. The association of rs2274110 genotype with tumor stage in the discovery, replication and combined ESCC samples** | | | | | | | | | | |
| --- | --- | --- | --- | --- | --- | --- | --- | --- | --- | --- |
|  | | **Discovery stage (*N* = 904)** | | | **Replication stage (*N* = 503)** | | | **Combined stages (*N* = 1407)** | | |
|  |  | **rs2274110 genotype** | | ***P*^†^** | **rs2274110 genotype** | | ***P*^†^** | **rs2274110 genotype** | | ***P*^†^** |
|  |  | **AA** | **AG+GG** |  | **AA** | **AG+GG** |  | **AA** | **AG+GG** |  |
| Tumor stage | |  |  | 0.0034 |  |  | 0.0016 |  |  | <0.0001 |
|  | I | 59 (7.8) | 23 (15.2) |  | 28 (6.7) | 14 (16.9) |  | 87 (7.4) | 37 (15.8) |  |
|  | II | 285 (37.8) | 67 (44.4) |  | 139 (33.1) | 35 (42.2) |  | 424 (36.1) | 102 (43.6) |  |
|  | III | 346 (45.9) | 51 (33.8) |  | 209 (49.8) | 30 (36.1) |  | 555 (47.3) | 81 (34.6) |  |
|  | IV | 63 (8.4) | 10 (6.6) |  | 44 (10.5) | 4 (4.8) |  | 107 (9.1) | 14 (6.0) |  |
| ***^†^*** *P* values were calculated by Pearson x^2^ test. | | | | | | | | | | |

**Supplementary Figures:**


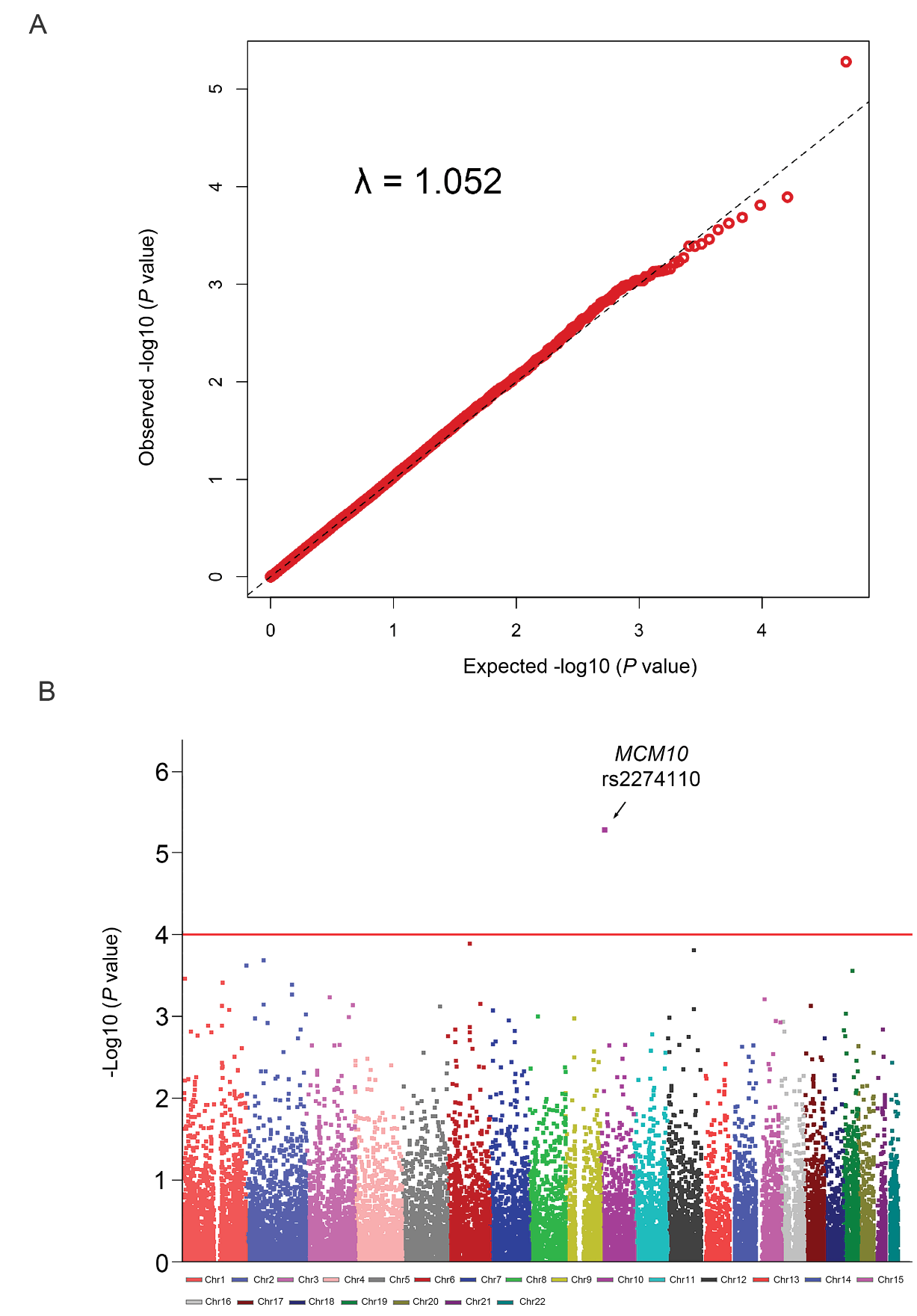


**Figure S1. Quantile-quantile plot and genomic inflation factor lambda for associations with ESCC prognosis.**

**A.** The results were based on 904 individuals with ESCC in the discovery stage of this study. The red circles represent the distribution of *P* values for the association in the discovery stage. The observed versus expected χ^2^ test statistics shows no evidence for inflation of χ^2^ tests (inflation factor λ = 1.052). **B.** Manhattan plot for associations between genetic variants and ESCC prognosis. The association analyses were based on 904 individuals with ESCC in the discovery stage of this study. *P* values were calculated by using a cox regression analysis with adjustments for age, gender, smoking status, drinking status and tumor stage. The associations (–log10 (*P*) values, y axis) are plotted against genomic position (x axis by chromosome and chromosomal position of NCBI build 37).


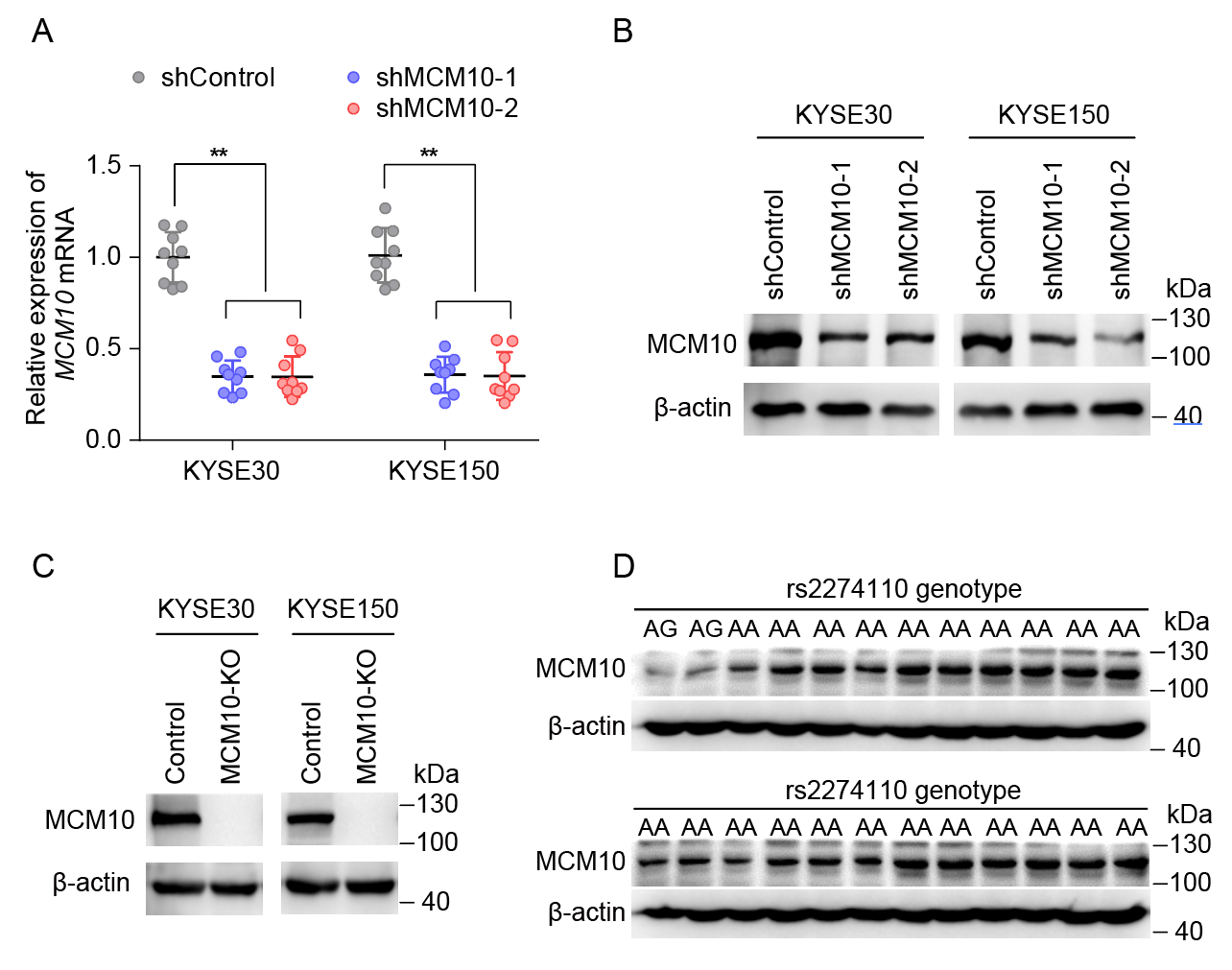
**Figure S2.** **Determination of the transfection efficiency**

**A, B.** Level of *MCM10* was tested by qRT-PCR (A) and western blotting (B) in KYSE30 and KYSE150 cells with *MCM10* knockdown using shRNA interfering. **C.** The efficiency of *MCM10* knockout was tested by western blotting using CRISPR/Cas9 method. **D.** The effect of variant rs2274110 genotype on MCM10 protein expression in ESCC samples using western blotting. Data in figure S2A were presented as the mean ± SD from three repeated experiments, each with triplicates and all ***P* < 0.01 were calculated by a two-sided Student’s *t*-test.


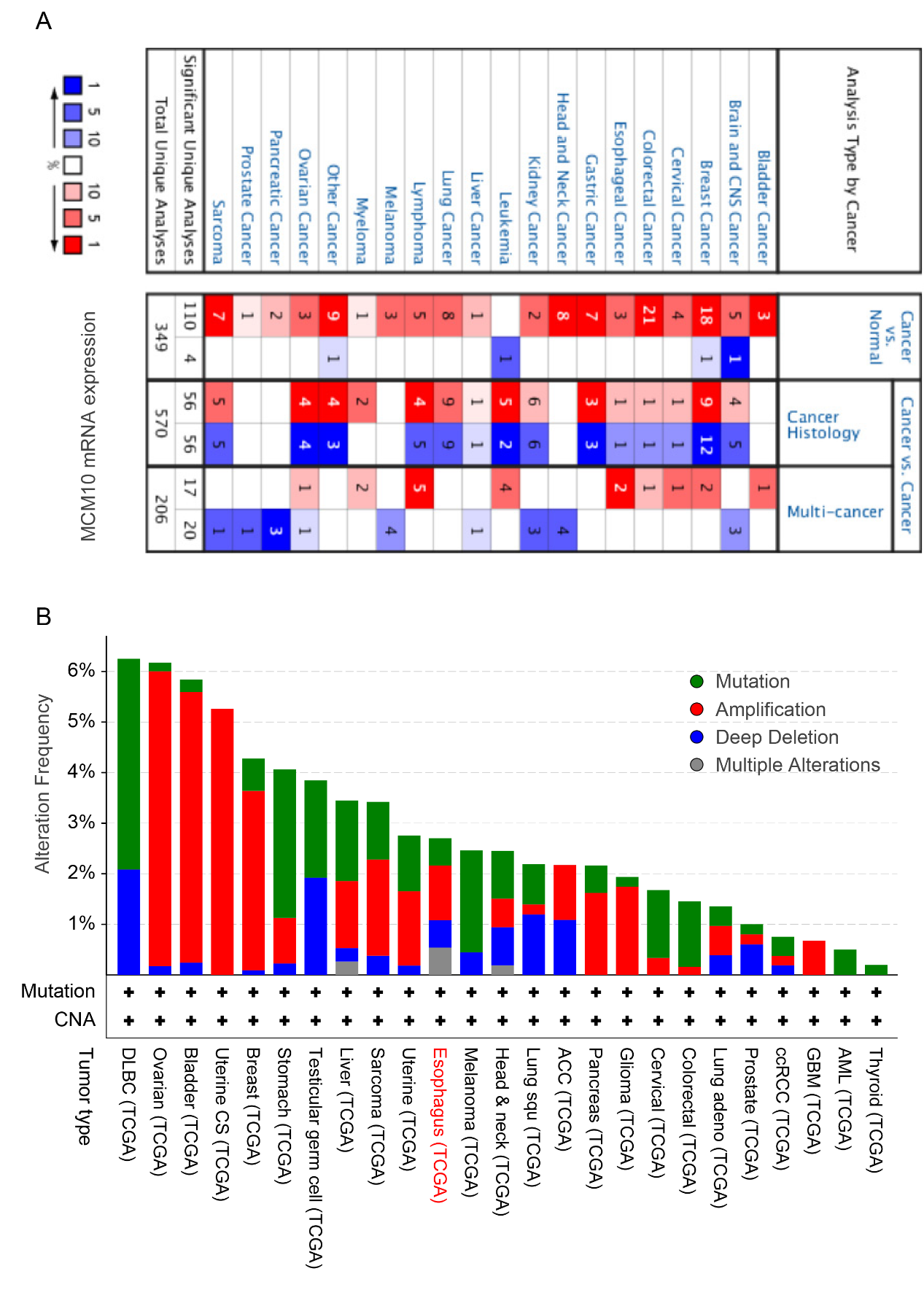


**Figure S3. MCM10 plays an important role in the ESCC**

**A.** *MCM10* expression levels were evaluated in multiple tumor types from the “Oncomine” database (https://www.oncomine.org). **B.** Genomic alterations and amplification of *MCM10* across multiple tumor types. The data were displayed as a histogram from the “cBioPortal” database (http://cbioportal.org). Esophagus (TCGA) indicates esophageal carcinoma.


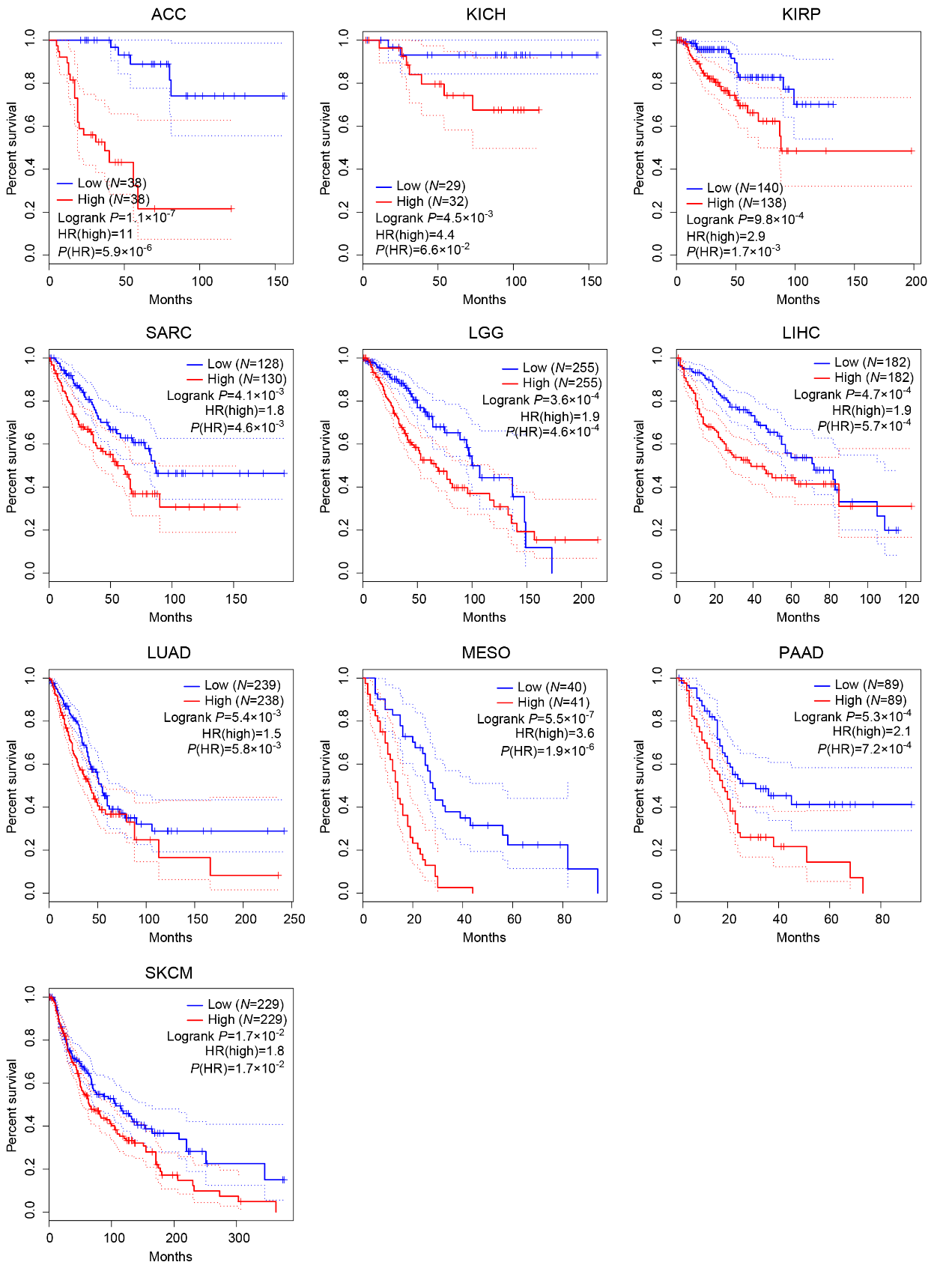


**Figure S4.** **The significant associations between high *MCM10* expression and poor survival in multiple cancer types.** Kaplan-Meier estimates of survival time for individuals in multiple tumor types, respectively, stratified by *MCM10* expression from TCGA. Logrank *P* values were calculated by the log-rank test, and hazard ratios (HRs) and *P*(HR) values were calculated using Cox regression analysis. ACC, Adrenocortical carcinoma; KICH, Kidney chromophobe; KIRP, Kidney renal papillary cell carcinoma; SARC, Sarcoma; LGG, Lower grade glioma; LIHC, Liver hepatocellular carcinoma; LUAD, Lung adenocarcinoma; MESO, Mesothelioma; PAAD, Pancreatic adenocarcinoma; SKCM, Skin cutaneous melanoma.
